# Nitrogenous Fertilizer Reduces Resistance but Enhances Tolerance to the Brown Planthopper in Fast-Growing, Moderately Resistant Rice

**DOI:** 10.3390/insects12110989

**Published:** 2021-11-03

**Authors:** Finbarr G. Horgan, Thais Fernanda S. de Freitas, Eduardo Crisol-Martínez, Enrique A. Mundaca, Carmencita C. Bernal

**Affiliations:** 1 EcoLaVerna Integral Restoration Ecology, Bridestown, Kildinan, T56 P499 Cork, Ireland; eduardocrisol@googlemail.com; 2 Escuela de Agronomía, Facultad de Ciencias Agrarias y Forestales, Universidad Católica del Maule, Casilla 7-D, Curicó 3349001, Chile; emundaca@gmail.com; 3 Plant Protection Department, Universidade Federal do Pampa, Itaqui 97650-000, RS, Brazil; thaisfs@gmail.com; 4 International Rice Research Institute, Makati 1226, Manila, Philippines; c.bernal@irri.org; 5 Association of Fruit and Vegetable Growers of Almeria (COEXPHAL), Carretera de Ronda 11, 04004 Almeria, Spain

**Keywords:** *BPH3*, *BPH32*, compensation, *Nilaparvata lugens*, phenotyping, phloem feeding, *Sogatella furcifera*, sustainable agriculture, xylem

## Abstract

**Simple Summary:**

The brown planthopper is a major rice pest in Asia that causes extensive damage where farmers apply large amounts of nitrogenous fertilizers. Over the last 50 years, host-plant resistance, i.e., a plant’s ability to deter planthoppers or reduce their survival and reproduction, has been the main focus of public research into planthopper management. However, there have been calls for increased attention to issues of rice tolerance against the planthopper, i.e., a plant’s ability to withstand attack and compensate for damage. We examined the interactions between planthoppers and 16 varieties of rice under varying levels of soil nitrogen. The varieties included susceptible, resistant and tolerant lines. We found that nitrogen generally increased planthopper fitness across the varieties; however, relative resistance was maintained in varieties with major resistance genes. Functional plant loss was found to be greatest in susceptible varieties after planthopper attack, and generally declined in resistant and tolerant varieties under increasing nitrogen levels. This increase in tolerance was most apparent among resistant and moderately resistant, large-sized, fast-growing varieties that were capable of overcoming relatively high weight and growth rate reductions.

**Abstract:**

The brown planthopper, *Nilaparvata lugens* (Stål), is a key challenge to rice production in Asia. Outbreaks of planthoppers are associated with excessive fertilizer applications; consequently, we examined planthopper interactions with susceptible, tolerant and resistant varieties of rice under varying levels of soil nitrogen in a greenhouse experiment. We compared planthopper fitness (survival × reproduction) and plant tolerance (functional plant loss index) for 16 varieties at 0, 80 and 150 Kg added nitrogen ha^−1^. The planthoppers grew larger, developed more quickly and laid more eggs on susceptible varieties, compared with the resistant and tolerant varieties. Moreover, soil nitrogen generally increased planthopper fitness on resistant varieties, but relative resistance was maintained. Functional plant loss was highest among the susceptible varieties, but weight and growth rate reductions per mg of planthopper were often highest in the tolerant varieties. Tolerance was associated with large, fast-growing plants, with at least moderate resistance to the planthopper. Susceptibility was associated with a small size and/or an absence of resistance genes. Our results suggested that early-tillering rice plants can be both resistant and tolerant to the brown planthopper, but cannot be both susceptible and tolerant of planthoppers at high densities. This indicates that at least moderate resistance is required for tolerance against this herbivore. Furthermore, although dwarf varieties had a low tolerance of planthoppers, they could express resistance through functioning resistance genes.

## 1. Introduction

Resistance can be described as a plant’s capacity to repel attack and reduce damage from herbivores [1,2,3]. Over the geographical range of a plant species, individual plants vary in their expressed resistance against specialized insect herbivores [4,5]. Resistance also varies between different plant tissues and over the course of plant growth and development [6,7]. Furthermore, plants that share the same nuclear genome (e.g., clones and pure-line crop varieties) may express different levels of resistance against herbivores depending on the plant’s environmental and growth conditions, particularly as this relates to resource availability (e.g., light, space, water and nutrients) [8,9,10], or the effects of other biotic stresses (i.e., infestation by other herbivores or diseases) [11,12,13,14,15]. The interactions between a plant phenotype and its environment in determining the levels of anti-herbivore resistance are particularly challenging for crop production. There are many well-documented cases of high fertilizer levels increasing plant susceptibility to herbivores. Such increased susceptibility can be due to changes in the plant’s antixenotic defenses (i.e., the plant’s ability to deter oviposition or herbivore settling), or its antibiotic defenses (i.e., the ability of the plant to reduce herbivore survival and reproductive output) [10,16]. For example, high nitrogen makes plants more attractive to ovipositing beetles and moths due to reduced antixenosis, and results in a greater level of progeny biomass (antibiosis) [17,18].

Resource availability can also alter a plant’s tolerance to herbivory [19,20]. Tolerance has been regarded as a component of resistance; however, it is now more commonly regarded as a separate trait that is largely determined by the plant’s physiological responses to stresses, by plant anatomy and size, and by the availability of resources for plant development [2]. Tolerance is a particularly beneficial trait for cereal farmers because many cereal crops have modular growth and can shift the distribution of resources between growing modules such as tillers, or between different plant organs, such as between roots, shoots and grain [19,21,22]. There are numerous well-documented examples of tolerance in cereal crops; for example, rice attacked by certain stem-boring moths can direct resources from the roots to undamaged tillers, to restore grain yields [22]. There are also several examples of the enhancement of tolerance through the addition of soil nutrients, such as nitrogenous fertilizers that enhance plant growth [18,23]. Despite the benefits and utility of plant tolerance, the trait continues to receive only scant attention from cereal breeders. Most research into plant–insect interactions continues to be directed towards susceptibility, i.e., the reduction of yields due to insects, and resistance, i.e., the search for major resistance genes against insects [24]. However, resistance and tolerance are not mutually exclusive; recently there have been calls for greater research attention, and further in-field applications of crop tolerance for a more sustainable management of insect herbivores [2,25,26].

Rice is a tillering plant with relatively rapid growth, particularly in tropical environments, and therefore rice plants often display a high tolerance to herbivory. Studies that have assessed rice weight losses or yield losses after attacks by herbivores have indicated that rice varieties vary considerably in their ability to withstand or compensate for damage [19,20,27,28,29,30,31]. Notably, some studies have indicated that certain rice varieties can overcompensate for damage and ultimately gain yield, particularly when attacked by stemborers or the white-backed planthopper, *Sogatella furcifera* Horváth [19,32]. Rice often gains tolerance as it ages and, moreover, tolerance can be enhanced when rice receives added nutrients, particularly nitrogenous fertilizers [19,20,33]. However, recent studies have indicated that for some herbivores, adding nitrogen to rice will accelerate herbivore damage if the extra nutrients benefit the herbivore more than the host plant [19,34]. The brown planthopper, *Nilaparvata lugens* (Stål), has long been recognized as a species that reaches outbreak densities where rice farmers use excessive nitrogen [35,36]. However, Horgan et al. (2018) [20] found that the addition of nitrogen could enhance tolerance in rice plants that have strong resistance to planthoppers. Although the added nitrogen increased the relative tolerance of the susceptible rice, it also enhanced planthopper population development, such that the planthoppers eventually killed up to 80% of the susceptible rice plants in field cages (i.e., where natural enemies were excluded). In their study, Horgan et al. (2018) [20] used only two rice varieties (one susceptible and one resistant); therefore, it is not known whether the effects of adding nitrogen to resistant rice were specific to the resistant variety used in their study (i.e., IR62), or are more widely observed among resistant rice in general.

In this study, we examine the effects of nitrogenous fertilizer on rice with a range of different responses to the brown planthopper. These included rice varieties without resistance genes that are therefore susceptible to planthoppers; varieties with resistance genes against which planthoppers have adapted, and are therefore now also susceptible; varieties with resistance to planthoppers because they possess functioning resistance genes, and varieties with observed tolerance to planthoppers without apparent major resistance genes. Among the potentially tolerant varieties, we included two hybrid varieties with heterosis for growth. We predicted that the addition of nitrogenous fertilizer would result in a greater fitness of planthoppers in all varieties (i.e., reduced resistance), but that the fitness gains would be lower for planthoppers on resistant varieties compared with susceptible and tolerant varieties. We also predicted that nitrogenous fertilizer would increase rice tolerance in plants with functioning resistance (i.e., with genes against which the planthopper has not yet adapted) and in tolerant varieties, but that susceptible plants would not gain a tolerance advantage from the extra nutrients. We further examined the plant growth rates and changes in growth after the infestation to determine the possible traits associated with tolerance. To our knowledge, this study includes the largest collection of rice varieties with distinct resistance genes (i.e., at least nine resistance genes) to be assessed for responses to fertilizer. We discuss our results in terms of phenotyping for resistance and tolerance, and for the effective use of resistant varieties in sustainable rice production systems.

## 2. Materials and Methods

### 2.1. Plant Materials

We used five varieties with high susceptibility to the brown planthopper. These included Taichung Native 1 (henceforth TN1) which is a *japonica* rice variety (the only one in our study) that is often used as a susceptible check in research on planthopper resistance in much of tropical Asia. The variety was first released in 1960 in Taiwan [29,37]. We also used the variety IR22 that was released by the International Rice Research Institute (IRRI) in 1969 and has no known resistance genes [38]. We used TKM6, a landrace from India thought to possess the *Bph1* gene for planthopper resistance, albeit in an inhibited state [39], ASD7 (Ac. 6303) with the *bph2* resistance gene [39], and IR46 and IR40, both developed at IRRI and with the *Bph1* and *bph2* genes, respectively [38]. Studies have shown that planthoppers throughout South and Southeast Asia are highly virulent against the *Bph1* and *bph2* genes, and therefore these latter four varieties are now susceptible to the planthopper [37,40]. As varieties with resistance, we used Swarnalata (Ac. 33964) with the *Bph6* gene [41], Pokkali (Ac. 15602) with the *Bph9* gene [42], ADR52 (Ac. 40638) with the *BPH25* and *BPH26* genes among other unidentified resistance genes [43,44], and IR65482-4-136-2-2 (IRTP 19029) (henceforth IR65482-4), a line with the *Bph10* gene introgressed from *Oryza australiensis*—a wild rice species from Australia [45]. We also used IR60 and IR62, released by IRRI in 1983 and 1984, respectively, both of which are thought to possess the *BPH3* and/or *BPH32* genes for resistance from the donor variety PTB33 [38,46]. The variety IR60 also has relatively high antixenosis/antibiosis against ovipositing planthoppers [47]. We used Triveni (Ac. 14785) and Utri Rajapan (Ac. 16684), landraces from India and Indonesia, respectively, with suggested tolerance to the brown planthopper [27,48,49]. We also used two hybrid rice lines, IR82396 H and IR82391 H from IRRI’s hybrid rice breeding program. IR82396 H has IR46 as a restorer line, and both hybrids were part of a previous study that showed enhanced tolerance to herbivores in hybrid rice under high nitrogen [19]. IR46 also displays a high level of tolerance to planthoppers in greenhouse and field trials [27].

### 2.2. Planthoppers

The planthoppers were obtained from a colony held at the Entomology Unit of IRRI. The colony had been initiated using >500 planthoppers collected in 2009, using sweep-nets in rice fields of the southern Laguna Province, Philippines (14°10′ N, 11°13′ E). The colony has been maintained for over 60 generations. The colony has also received periodic introgressions of wild-caught planthoppers from IRRI rice fields following monitoring, to indicate that individuals did not transmit rice viruses (i.e., no symptoms of Rice Grassy Stunt Virus [RGSV] or Rice Ragged Stunt Virus [RRSV] were detected during the initial generations). The planthoppers were reared on TN1 at >30 days after sowing (DAS) in wire mesh cages (92 × 57 × 57 cm: H × W × L) in a screenhouse. Their feeding plants were changed every week. The screenhouse temperatures ranged from 26–37 °C and humidity ranged from 70–90%. The screenhouse received natural lighting (12D:12N photoperiod). The colony had been screened for virulence using several bioassays (i.e., seedbox tests and bionomic tests) and was shown to be virulent against varieties with the *Bph1*, *bph2*, *bph5*, *bph7*, *Bph18*, *BPH25*, and *BPH26* genes [40].

### 2.3. Effects of Nitrogen on Rice–Planthopper Interactions

Rice seed of each variety was initially planted in plastic trays, with one variety per tray, in moistened paddy soil. At 7 DAS, seedlings of each variety were transferred to #0 pots (7 × 11 cm: H × diam) with paddy soil. The pots were maintained on a greenhouse bench and were watered and weeded daily. The plants received no pesticide treatments, and the seedlings were allowed to grow and develop for a further 14 days, after which the pots were divided into three groups. The plants received one of three fertilizer treatments: zero added fertilizer, fertilizer added equivalent to 80 Kg N ha^−1^, or fertilizer equivalent to 150 Kg N ha^−1^. The high nitrogen level was selected to simulate high inputs, as reported from farmers’ fields in Asia [8]. The medium level was based on an analysis of nutrient requirements using a Rice Crop Manager [20] at the rice paddy site where the soil was originally collected. The levels of plant-available nitrogen were not monitored after the fertilizer applications. Nitrogen was added to the pots as ammonium sulphate, with two thirds added (groups 2 and 3) at 21 DAS and the remainder at 28 DAS. This simulated the field conditions where nitrogen is added basally, or at the early crop stages before planthoppers arrive to the fields, and again during the crop stages when plants have become infested with planthoppers [8,19,20]. Individual plants were used in three separate bioassays, as described below.

Firstly, we used the honeydew test. In this process, individual plants of each variety were passed through a plastic feeding chamber (5 × 5: H × diam) placed directly over the potted soil at 30 DAS. Each chamber had a hole at the base and top, through which the rice plant passed. A Whatman no. 1 filter paper treated with bromocresol green was placed at the base of each chamber. Bromocresol green indicates phloem-derived honeydew as blue-rimmed spots and xylem-derived honeydew as white-rimmed spots. Two gravid, female planthoppers (pre-oviposition) were placed in each chamber through the top hole. We used gravid females because these were relatively large compared with the other planthoppers and produced larger quantities of honeydew. After adding the females to each chamber, the hole was sealed with a cotton plug. The planthoppers were allowed to feed for 24 h, after which they were checked to verify that both planthoppers were still alive, and the filter papers were collected. The papers were then digitized, and the areas of excreted honeydew were measured using Image J version 1.48 (National Institute of Health, MD, USA). The experiment was set up on a laboratory bench (T = 25–27 °C, RH = 70–75%, 12D:12N) as a randomized block design, with three replicated blocks (16 varieties × 3 nitrogen levels × 3 replicates = 144 pots).

Next, we performed the Survival test. After the final fertilizer addition at 28 DAS, each plant was placed under an acetate cage (61 × 10 cm: H × diam) with a mesh top. At 30 DAS, three plants of each variety were infested with eight newly hatched planthoppers per plant. The planthoppers, obtained from the colony using a pooter, were placed inside the acetate cages through a slit in the cage wall. Additionally, a set of non-infested cages with plants at the same age as the infested plants were maintained as controls. Half of the control plants of each variety were destructively harvested at 30 DAS to estimate the plant biomass at the beginning of the experiment. The plants were harvested by being pulled from the moistened soil, after which their roots were carefully washed under running water. The plants were separated into above-ground shoots and below-ground roots, with the shoots and roots placed in separate bags (i.e., two bags for each sample). The remaining plants (controls and infested) were maintained on benches inside three separate greenhouse compartments (separated by insect-proof screening). Each compartment represented a replicated block (16 varieties × 3 nitrogen levels × 2 control/infested treatments × 3 replicates = 288 pots).

After 15 days, when the plants were at 45 DAS, they were rated for damage using the standard evaluation system (SES) [50] and the planthoppers were collected from the infested cages using a suction sampler. All plants were then destructively harvested (as described above) with the roots and shoots from each plant placed in separate bags. The planthoppers and rice plants were placed in a forced draft oven at 60 °C for 7 days, after which they were weighed on a precision balance. The planthoppers were then examined under a stereo microscope (10× magnification) to determine the developmental stage of each planthopper and, where adults had emerged, were sexed, and the wing forms recorded as brachypterous (short-winged) or macropterous (long-winged).

Finally, we utilized the oviposition test. At 30 DAS, three plants of each variety under acetate cages (61 × 10 cm: H × diam) were infested with recently emerged, previously non-mated planthopper pairs (1 male, 1 female). The plants were maintained on three greenhouse benches, each inside a separate greenhouse compartment, representing three replicated blocks (16 varieties × 3 nitrogen levels × 3 replicates = 144 pots). The planthoppers were allowed to feed and mate, and the females were allowed to oviposit on the plants for 7 days, after which time the adults were removed. The plants were then destructively harvested as described above and placed in a refrigerator (4 °C), with each plant wrapped in moistened tissue paper. Within 5 days, the plants were dissected under a stereo microscope (10× magnification) and the number of eggs in each plant were recorded.

### 2.4. Data Analyses

The proportion of total honeydew that was derived from xylem feeding was used as an indicator of host resistance. This measure standardized the estimates of honeydew production for potential variability in adult size. We examined the honeydew production from the honeydew test; the final nymph biomass; the proportion of nymphs at the adult stage; the proportion of adults that were female; the proportions of adult females and males that were brachypterous from the Survival test, and the number of eggs laid per plant from the Oviposition test using univariate general linear models (GLM). We included block as a random variable and initially included plant weight (at 30 DAS) as a covariate in each test, removing the covariate where there was no significant effect. We examined the linear contrasts associated with the nitrogen effect. Post-hoc tests were conducted using Tukey’s pairwise comparisons and Duncan’s many-to-one tests against TN1 as a standard susceptible check. The nymph biomass was log-transformed before analysis, and the any proportions (xylem, adults, females, brachypterous forms) were arcsine-transformed before analyses took place. Data residuals were plotted following all analyses and were found to be normal and homogenous.

We used repeated measure GLMs to analyze the shoot and root biomass. The plant growth rates were estimated as the difference between total plant biomass at 45 DAS and 30 DAS, divided by time (=15 days). Plant growth rates were compared using a univariate GLM with the initial plant weight (i.e., at 30 DAS) as a covariate. To examine the effects of planthoppers on the host plants, we first estimated the plant weight loss difference between infested plants and non-infested plants at 45 DAS, and estimated the weight loss per unit of insect weight by dividing this number by the weight of the planthoppers on the same plants (Schweissing and Wilde 1978 [27]). For the damaged plants, the remaining biomass could have lost functionality (e.g., loss of chlorophyl content, loss of water content [51]), and thus we also calculated the functional plant loss index (FPLI) using the method described by Panda and Heinrichs (1983) [27]. The FPLI is calculated as the product of proportional plant weight loss and proportional damage to the plant. The proportional damage was calculated as the SES damage rating for each plant at the end of the Survival test, divided by 9—the damage rating assigned to herbivore-killed plants. FPLI is expressed as a percentage reduction in functionality. Plant weight loss, the loss in plant weight per mg of planthopper, and FPLIs were each analyzed using a univariate GLM with the initial plant weight as a covariate. Linear contrasts were examined for nitrogen effects, and data residuals were plotted following analyses that were found to be normal and homogenous.

Based on the parameters obtained via the different bioassays, rice lines were compared using the permutational multivariate analysis of variance (PERMANOVA) [52] on Euclidean distances calculated from normalized data. The PERMANOVA analysis included two factors; nitrogen, with three levels (1 = zero added nitrogen, 2 = 80 Kg ha^−1^, 3 = 150 Kg ha^−1^), and resistance, with two levels (whether the rice line had functioning resistance genes (i.e., other than *Bph1*, *bph2*, *BPH25* or *BPH26*) [R] or was without major functioning resistance genes (i.e., susceptible) [S]). A canonical analysis of principal coordinates (CAP) was used to visualize the differences between the groups of rice lines [53], based on the parameters obtained from the plant–insect bioassays. All multivariate analyses were conducted using PRIMER-E version 6.1.16 software with the PERMANOVA+ add-on (v. 1.0.6).

## 3. Results

### 3.1. Host Resistance

Planthoppers on nine of the rice varieties produced more xylem-derived honeydew compared to planthoppers on TN1. These nine varieties included all varieties with the *Bph3/Bph32*, *Bph6*, *Bph9* and *Bph10* genes. The variety ADR52 also appeared more resistant that TN1 based on xylem-derived honeydew (Table 1). Nitrogen as a main factor did not affect honeydew composition; however, there was a significant linear decline in xylem-derived honeydew with increasing nitrogen (linear contrast: *p* = 0.028) (Table 1).

Damage due to planthoppers in the Survival test (based on SES ratings) was lowest in IR62, IR60, ADR52, IR65482-4 and Triveni compared with the susceptible check TN1 (F_15,96_ = 16.913, *p* < 0.001). Damage increased with added nitrogen (F_2,96_ = 5.148, *p* = 0.008), however, there was a significant interaction (F_30,96_ = 1.729, *p* = 0.024) due to the decreasing damage to Pokkali and IR82396-4, and increasing damage to IR22 and IR40 under high nitrogen (Table S1). There was no variety effect on nymph survival (Table S1). Most nymphs were at the 5th instar stage or had reached the adult stage within 15 days (Table S1); however, nymphs had slower development on IR62 and ADR52 compared with TN1 (Table 1). Variety had no effect on the proportion of adults that were female (Table 1) or the proportion of females that were brachypterous (Table S1). More males were brachypterous on IR62 and TN1 compared to all other varieties (Table S1). Nymphs that developed on IR62 (*Bph3/Bph32*), ADR52 (unknown genes), IR60 (*Bph3/Bph32*), IR65482-4 (*Bph10*) and Pokkali (*Bph9*) had a lower biomass than the nymphs on TN1, but only nymphs on IR65482-4, IR60 and IR62 were smaller than nymphs on IR22 (Table 1). The nitrogen level did not affect survival rates, nymph weights, nymph development rates, or the proportions of males and females that were brachypterous in the bioassay (Table 1 and Table S1). Proportionally, fewer females emerged under high nitrogen (Table 1).

Planthoppers laid fewer eggs on 13 of the varieties compared with TN1 (Table 1). However, fewer eggs were laid only on IR60, Pokkali and Triveni, when compared with IR22 (Table 1). More eggs were laid on plants under high nitrogen as indicated by a significant linear contrast (*p* = 0.016). There was a significant variety × nitrogen interaction due to a higher number of eggs on TN1, IR22, TKM6, IR40, ASD7 and Utri Rajapan under zero added nitrogen when compared to other varieties at the same nitrogen level; however, planthoppers laid a similar number of eggs on all varieties under high nitrogen, except on Triveni, where egg numbers remained relatively low even under high nitrogen (Table 1).

### 3.2. Plant Biomass and Growth Rates

Non-infested plants gained shoot and root weight between 30 and 45 DAS (shoots: F_1,94_ = 1265.430, *p* < 0.001; roots: F_1,94_ = 688.561, *p* < 0.001) (Table S1). The IR varieties, including IR22, IR40 and IR62 and the hybrid IR82391 H had the lowest shoot weights, with Utri Rajapan, Triveni and ADR52 having the largest (Table 2). Similarly, IR22, IR40 and IR82391 H had low root biomass and Utri Rajapan and Triveni had relatively large roots (Table 2). TN1 plants were also relatively large (Table 2). Slower gains in shoot and root biomass in IR40, IR82391H, ASD7, and TKM6 when nitrogen was increased from 80 to 150 Kg ha^−1^, and the smaller size of IR22, IR40, and IR62 compared with Utri Rajapan and Triveni, produced significant time × nitrogen (shoots: F_2,94_ = 82.866, *p* < 0.001; roots F_2,94_ = 23.265, *p* < 0.001) and time × variety interactions (shoots: F_15,94_ = 6.010, *p* < 0.001; roots F_15,94_ = 4.458, *p* < 0.001) (Table 2). Over the course of 15 days, non-infested Utri Rajapan, Triveni, ADR52, TN1, and IR82396 H plants had the fastest growth rates and grew faster than IR40 and IR82391 H (Table 2). These growth rates were significantly related to the initial sizes of the plants (covariate 30 DAS plant biomass: F_1,94_ = 306.357, *p* < 0.001). Roots and shoots increased with increasing nitrogen, and growth rates were higher as fertilizer levels increased (linear contrast: *p* < 0.001: Table 2).

### 3.3. Planthopper Effects on Plants

The IR40 and IR82391 H plants lost the least amount of weight after infestation, and lost significantly less weight than Utri Rajapan and IR82396 H (Table 2). Weight loss was positively affected by the initial plant weights (covariate: F_1,94_ = 106.449, *p* < 0.001) and increased with increasing nitrogen levels (linear contrast: *p* < 0.001: Table 2). Weight losses per unit of insect weight were the lowest on IR22, IR40, TKM6, ASD7 and IR83291 H, and were significantly lower than the losses in Triveni (Table 2). The covariate plant weight also positively affected weight loss per mg of insect (F_1,94_ = 25.405, *p* < 0.001), indicating that larger plants lost more weight over the course of the bioassay. The weight loss per mg of insect increased with increasing nitrogen levels (linear contrast = 0.001: Table 2).

**Figure 1:**
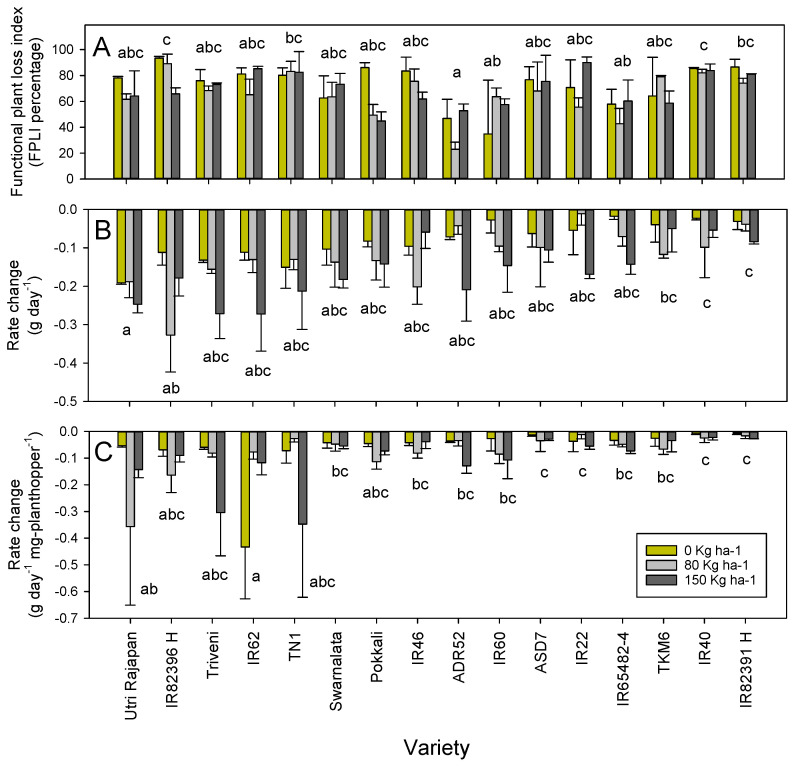
(**A**) FPLI for 16 varieties/lines at three nitrogen levels, with (**B**) changes in plant growth rate between 30 and 45 DAS under three levels of soil nitrogen after infestation with planthopper nymphs and (**C**) corresponding growth rate changes per mg of planthopper. Error bars are indicated (N = 3). Lowercase letters indicate homogenous variety groups (Tukey *p* > 0.05).

The FPLI indices were lowest for two relatively large plants with resistance genes (ADR52 and IR65482-4) and were greatest for the hybrid rice lines, and for IR40 and TN1 (F_15,96_ = 3.241, *p* < 0.001: Figure 1A). The introduction of nitrogen generally reduced FPLI (F_2,96_ = 4.953, *p* = 0.009: Figure 1A), indicating a nitrogen-induced increase in tolerance to the planthoppers. The effects were most pronounced for IR46, and for IR82396 H, which has IR46 as a restorer and likely contained the *Bph1* gene, and for Pokkali, which possesses the *Bph9* gene. The interactions were not significant.

Plant growth rates were reduced by planthopper feeding. The rate reductions were greater in Utri Rajapan and IR82396 H compared with IR40 and IR82391 H (F_15,94_ = 3.665, *p* < 0.001: Figure 1B) and rate reductions increased under increasing nitrogen (F_2,94_ = 10.130, *p* < 0.001, linear contrast = 0.001: Figure 1B). The plant growth rate reduction per mg of insect was greatest in IR62 and Utri Rajapan, and greater than reductions in IR22, IR40, IR82391 H, and ASD7 (F_15,96_ = 1.992, *p* = 0.024: Figure 1C). Nitrogen did not affect the rate reductions per mg of insect (F_2,94_ = 1.229, *p* = 0.297: Figure 1C). The interactions were not significant.

**Figure 2:**
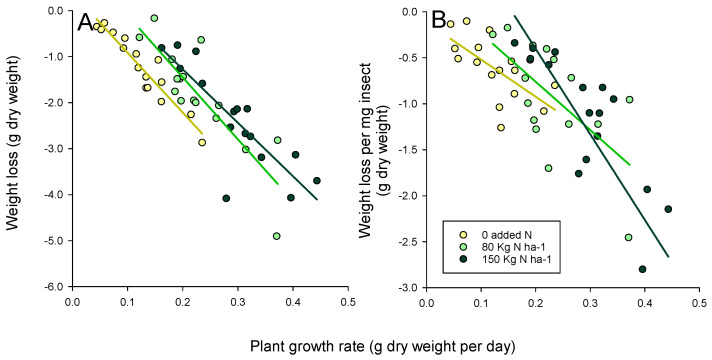
Relations between plant growth rate and (**A**) plant weight loss, and (**B**) weight loss per mg of planthopper for 16 rice varieties exposed to planthoppers under three nitrogenous fertilizer regimes (see legend). Each point is the average of three replicates. Best-fit linear models are indicated for each nitrogen level (Table 3).

The plants with faster growth rates lost greater amounts of biomass in the bioassays and had greater weight losses per mg of insect (Figure 2). The slopes for weight reductions per mg of insect tended to be greater under high nitrogen (Table 3), indicating greater tissue losses to plants under high nitrogen; however, the slopes of the relations were not statistically significantly affected by the nitrogen levels (weight loss: F_2,6_ = 0.418, *p* = 0.676; weight loss per mg insect: F_2,6_ = 0.769, *p* = 0.504: Figure 2).

The PERMANOVA analysis showed significant effects of nitrogen (Pseudo-F = 4.318, *p* = 0.001) and resistance (Pseudo-F = 9.901, *p* = 0.001) on the multivariate comparison of rice plants. The interaction term ‘nitrogen × resistance’ was not significant (Pseudo-F = 1.178, *p* = 0.260), indicating that the possession of a functioning resistance gene did not generally determine planthopper–rice interactions under varying nitrogen levels.

**Figure 3:**
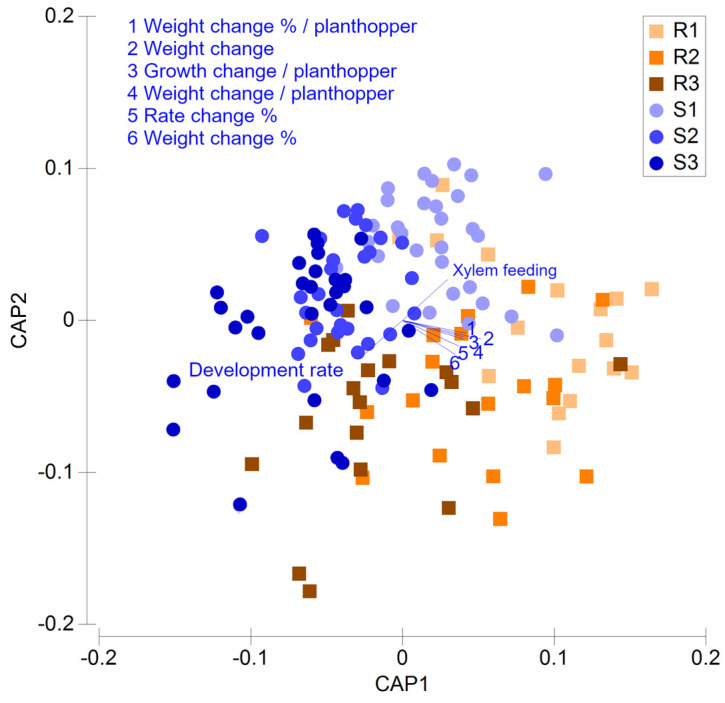
Canonical Analysis of Principal Coordinates (CAP) depicting differences in herbivore fitness and plant damage parameters obtained through multiple bioassays. Squares represent resistant varieties/lines, whereas circles represent susceptible varieties/lines. Nitrogen regimes (1 = 0 added N, 2 = 80 Kg N ha^−1^, and 3 = 150 Kg N ha^−1^) are represented by increasing color darkness. Overlayed vectors (in blue) represent the correlations (Pearson’s correlation coefficient > 0.6) between rice lines and the CAP axes, where vector length and direction reflect the increasing values of correlation and parameter values, respectively.

The CAP plot (Figure 3) largely separates resistant (i.e., with functioning resistance genes) from susceptible varieties based on high proportions of xylem feeding and the slower development from nymph to adult on the resistant varieties. Relatively high xylem feeding and slower development to adults were also associated with lower amounts of nitrogenous fertilizer. Triveni, Utri Rajapan, IR46 and IR82396 H overlapped with the resistant varieties because of relatively slow development and high xylem feeding under one or other nitrogen treatment. Greater plant-weight and growth-rate losses, and greater losses per mg of planthopper were observed among the varieties that lacked identified functioning resistance genes, without a strong effect of nitrogen.

Overall, the CAP plot indicated that interactions between planthoppers and tolerant varieties were often more similar to interactions between planthoppers and resistant varieties under low or high nitrogen levels (depending on the tolerant variety in question).

## 4. Discussion

Several studies have examined the effects of plant and soil nitrogen on development and population increases in the brown planthopper [19,20,34,35,36,43,47,54,55,56,57,58,59]. The planthopper appears to have a relatively high demand for nitrogen compared with other rice herbivores and, unlike other herbivores, will more frequently kill susceptible rice plants under high nitrogen conditions than under low nitrogen conditions [19,20]. Studies have shown that planthopper feeding reduces the content of nitrogen in the plant tissues and reduces nitrogen assimilation through the plant roots, although these effects will differ depending on the rice variety [55,60,61,62]. Furthermore, increasing the levels of nitrogenous fertilizer in the soil reduces resistance in rice, where resistance is measured using planthopper fitness parameters [8,20,36,47]. Our study used a large range of plants that included susceptible, resistant and tolerant varieties to further examine this effect.

### 4.1. Effects of Adding Nitrogen on Planthopper Fitness

We predicted that although resistance would decline under high nitrogen, those varieties with functioning resistance genes (i.e., genes against which planthoppers have not yet adapted) would remain relatively resistant compared with the standard susceptible check varieties such as TN1. We found that xylem feeding, which is an indicator of resistance, generally declined under increasing fertilizer levels. This trend was strong in susceptible varieties, but was weaker among the resistant and tolerant varieties, with xylem-derived honeydew increasing among planthoppers on some varieties, such as Pokkali and Triveni, under the higher nitrogen levels (Table 1). Compared with other planthoppers, i.e., the white-backed planthopper, xylem feeding is low when the brown planthopper feeds on susceptible varieties [47], indicating the high efficiency of the brown planthopper in tapping phloem sieves. The reasons for xylem feeding by planthoppers on resistant rice are still largely unknown; it has been suggested that planthoppers may feed on xylem to dilute plant toxins, or to rehydrate during periods of low food assimilation [63,64,65]. Xylem feeding may also result from the inability of planthoppers to successfully locate phloem tubes. However, Sharma et al. (2013) [58] have shown that planthopper probing declines significantly as nitrogen levels in the soil increase. Excessive probing is related to difficulty in finding phloem tubes, and therefore these observations suggest that the planthoppers encounter the tubes more successfully under high nitrogen.

More efficient feeding and improved food quality resulted in faster growth and development and larger individuals on the nitrogen-fertilized rice plants (Table 1). Egg laying also tended to increase as nitrogen levels increased, particularly on resistant plants (Table 1). High soil nitrogen has been associated with a decline in silicon content in rice plants, which may also contribute to a higher fitness of planthoppers, because silicon reduces feeding and development rates [34,66,67]. An increase in planthopper fitness in response to soil nitrogen was generally apparent in the present study (Table 1); however, although the interaction between varieties and nitrogen was not statistically significant, there was a tendency for development rates to increase for planthoppers on resistant and tolerant varieties, and to decline on susceptible varieties as the nitrogen rates increased (Table 1). This may be related to the smaller sizes of the planthoppers on the resistant and tolerant plants (i.e., tolerant plants were also relatively resistant to the planthopper) compared with those on susceptible plants, with larger planthoppers taking longer to develop. In the Survival bioassay, the SES damage ratings did suggest that susceptible plants under high nitrogen deteriorated more quickly than plants without added nitrogen (Table S2), which probably affected the magnitude of the nitrogen effect on planthopper fitness on susceptible plants. Despite this, as shown by the CAP plot, our results strongly indicated that rice varieties maintain their relative resistance or susceptibility irrespective of soil nitrogen levels (Figure 3). The ability of resistant plants to maintain their relative resistance (even though that resistance was weakened) corroborated with evidence from a review of previous studies that mainly included genes against which the brown planthopper has now adapted (i.e., *Bph1* and *bph2* [8]). Despite the maintenance of relative resistance under varying levels of soil nitrogen, actions to counter nitrogen-induced fitness gains on resistant rice are still required to avoid planthopper virulence adaptation. Furthermore, the resistance to specific herbivores can be compromised where plants are attacked by other herbivores or diseases, as has been shown in other crop–herbivore systems [11,12,13,14]. In rice, a prior attack by the white-backed planthopper can reduce fitness in the brown planthopper, thereby adding to the effectiveness of brown-planthopper-resistant rice [15]. In well managed rice production systems, specialist natural enemies such as *Cyrthorhinus lividipennis* are expected to maintain planthopper populations at relatively low densities, thereby countering any increased reproductive output on resistant rice, due to high nitrogen or to possible losses to host resistance resulting from other biotic and abiotic stresses [54,68]; however, where insecticides reduce natural enemy abundance, nitrogen-mediated reductions in resistance could lead to the increasingly rapid adaptation by planthoppers to the host’s resistance as populations build up in fields of resistant rice [8,35].

### 4.2. Effects of Adding Nitrogen on Rice Tolerance

Planthopper damage to rice appears as a loss of chlorophyll, a reduction in relative water content, a decline in plant-nitrogen content, and a decreased uptake of nitrogen from the soil [34,55,61,62,69,70]. This ultimately leads to a reduction in growth rates and a loss in final plant weight [19,20,21,27,71]. We predicted that the resistant plants would appear more tolerant of damage than the susceptible plants and that their tolerance would increase under high nitrogen. For example, high soil nitrogen has been associated with a higher tolerance in maize to stemborers, despite the declining resistance of the maize (i.e., improved stemborer fitness) under high nitrogen [23]. The effects of nitrogen on rice tolerance to herbivores have previously been examined for hybrid varieties and their parental lines in a greenhouse study, and in a comparative study of IR62 and IR22 using field cages. It was found that high nitrogen levels improved the hybrid tolerance against stemborers and the white-backed planthopper [19], and improved tolerance in the resistant variety IR62 to the brown planthopper [20]. The differences in tolerance responses to the different herbivores may be related to the high nitrogen assimilation efficiency of brown planthoppers; for example, planthoppers can recycle nitrogenous wastes through the actions of yeast-like symbionts in the insect gut [63,72,73].

We predicted that high nitrogen would prolong plant survival and largely maintain the weight increases of resistant and tolerant rice plants exposed to planthoppers, but would reduce the growth rates of susceptible varieties. Furthermore, we expected that the weight losses to rice plants per mg of planthopper would be greater for the susceptible varieties. Our results, based on FPLI, largely agreed with our predictions. The susceptible check TN1, the highly susceptible variety IR40, and the hybrid lines had among the highest FPLI indices. Meanwhile, the resistant varieties ADR52 and IR65482-4 had the lowest FPLI indices (Figure 1). We found that nitrogen reduced FPLI, with the strongest trends apparent with Pokkali, Utri Rajapan, IR46 and with the IR46-derived hybrid IR82396 H (Figure 1). However, when considering weight and growth rate changes alone, i.e., without regard to chlorophyll and condition losses, the results did not generally distinguish susceptible and resistant varieties. This differed from studies conducted using bulk tests [50,51], and tests conducted with larger, often older rice plants [19,20,21,27,29]. Despite these differences, our results, based on accumulated changes to plant weight and to growth rates, as well as changes per mg of planthopper, revealed the role of plant anatomy in determining apparent tolerance in early-tillering rice plants.

In general, the greatest losses to plant weight and growth rates in our bioassays were observed among the tolerant varieties Utri Rajapan, Triveni and IR82396 H, and in the resistant variety IR62. Furthermore, the weight and rate reductions were generally greater under higher nitrogen conditions (Table 2). These trends were principally due to the relatively large sizes of the tolerant varieties and the larger size of the nitrogen-fertilized plants, as revealed through a strong plant biomass effect (Table S2). Therefore, plant size may be a simple indicator of tolerance to planthoppers, with larger plants able to withstand greater damage. Conversely, small plants were unable to withstand damage, and were therefore unlikely to survive planthopper attacks unless they possessed strong resistance. For example, relative weight losses and rate reductions were similar for several resistant and susceptible plants in our study (i.e., ADR52, IR60, IR65482-4, Swarnalata, Pokkali, TKM6, and IR22: Table 2, Figure 1); however, damage ratings (i.e., losses to functional capacity) were greatest for the susceptible plants. This agrees with the observation that vigorous plants likely suffer greater levels of herbivory compared with less vigorous plants, at larger geographical scales [3,74]. Moreover, plant vigor is often related to growth rates. Our estimates of growth rates were based on the plant biomass at 30 and 45 DAS, but growth rates change over the course of plant development. We recommend that future studies of tolerance might gain increased accuracy by estimating growth rates between a larger number of rice development stages [75].

### 4.3. The Need to Combine Resistance with Tolerance

Plant tolerance is often examined using proportional losses to standardize for differences in plant size [19,27,76]; however, when examining absolute losses in this study, it became increasingly apparent that plant size plays a large role in tolerance and susceptibility, and that nitrogen-induced increases in plant size may partly explain the higher tolerance of some plants under high fertilizer regimes. For example, IR62 in both this study and a previous one [20] appears to have a high nitrogen-induced tolerance to planthoppers compared with, for example, IR60, despite both varieties having the same resistance sources and possibly the same resistance genes (*Bph3/Bph32*) (Figure 1). Furthermore, a smaller size prevents plants from withstanding planthopper attacks, and may be associated with high susceptibility. This has important implications, given that many modern varieties have been derived from crosses with dwarf and semi-dwarf varieties including Dee-geo-woo-gen, which possesses the *semidwarf-1* (*sd1*) gene [38]. Outbreaks of the brown planthopper in tropical Asia were first noted at the beginning of the Green Revolution (late 1960s–early 1970s) and had been associated with modern varieties of fertilizer and pesticides [77]. Our results suggested that dwarfism likely contributed to the high damage from planthoppers at that time—particularly where pesticides reduced the abundance of natural enemies—because small rice plants have little tolerance to attack and, at the time, were not yet bred for resistance. These ideas could be tested in future research using more detailed field experiments and a greater range of rice varieties.

Overall, our results suggested that resistance is a prerequisite for tolerance in small or dwarf rice varieties. Furthermore, large plants, such as many traditional varieties, will probably be more tolerant to planthopper damage. In our study, such traditional, tolerant varieties also appeared to have at least moderate resistance from unidentified genetic sources (Table 1). For example, TN1 is a relatively large plant that was released over 60 years ago [38], but is highly susceptible to planthoppers and therefore generally exhibits a high FPLI, making it a useful check variety in studies of planthopper resistance. Meanwhile Utri Rajapan and Triveni, both noted for their tolerance to planthoppers [27,48,49,78], were relatively resistant to ovipositing planthoppers and induced relatively high xylem-based feeding at low (Triveni and Utri Rajapan) or high (Triveni) nitrogen levels (Table 1). Tolerance could be enhanced by soil nitrogen, but this nitrogen-induced tolerance was also associated with larger plants, faster growth rates, and at least moderate resistance. Among resistant semidwarf varieties, IR62 appeared to possess a higher capacity for nitrogen-induction of tolerance, compared with other resistant varieties (i.e., IR60, IR65482-4) (Figure 1). Hybrid varieties were proposed to have a naturally higher tolerance to planthoppers compared with their parental lines, simply because of heterosis for growth rates [19,79,80]. However, our results indicated that hybrid tolerance must be enhanced by the careful selection of parental lines. For example, IR82396 H was more tolerant of planthopper damage under high nitrogen compared with IR82391 H. This higher tolerance of the former hybrid was likely related to IR46 being used to restore fertility during breeding; IR46 is a semidwarf variety with the *Bph1* resistance gene and possesses a previously reported level of tolerance to planthopper damage [27].

Although plant size is important, size is not the only feature of tolerance. Some plants can actively increase photosynthesis in non-damaged tissues, or shunt resources toward non-damaged tissues, including intact tillers. Plants may also alter their phenology in response to herbivore damage to better withstand attack [21,26]. Therefore, future breeding programs could screen lines for high tolerance based on these characteristics. An efficient screening for tolerance is difficult to achieve where large numbers of rice lines pass through breeding pipelines [50]. The methods used in this study were somewhat preliminary but have been successfully applied in previous studies to identify tolerance-related QTLs [29,76,81]. We suggest that for improved results, researchers should use large pots that do not inhibit plant growth, should assess tolerance at different crop stages [75], and should avoid intraspecific competition between planthoppers where the host quality is reduced because of damage [79]. Even where plants show no visible signs of damage, they may undergo physiological changes that otherwise reduce nutrient availability to conspecific planthoppers and increase intraspecific competition [51]. Furthermore, because tolerance can increase or decline under high nitrogen—depending on the nature of specific resistance genes and resistance mechanisms [8]—phenotyping for tolerance should normally be conducted using soil with added nitrogen, to better simulate paddy-field conditions.

Ensuring that rice plants have tolerance, as well as moderate resistance, may be essential for sustainable rice production. For example, where planthoppers adapt to resistance genes, damage to the now-unprotected plants could be severe unless the plants are also tolerant of damage. Furthermore, resistance is often unstable under varying weather and climatic conditions, or where plants are attacked by a community of herbivores [11,12,13,14,15], and damage could be intense for non-tolerant plants when resistance becomes ineffective, for example, during periods of high temperatures [82,83,84,85]. Indeed, Alam and Cohen (1997) [86] have indicated that the success of IR64, one of Asia’s mega-varieties, has been due to the variety’s high herbivory tolerance, despite widespread adaptation by the brown planthopper to its *Bph1*-based resistance. The promotion of larger plants through the careful choice of rice varieties, including hybrid varieties, and through increased plant spacing during sowing or transplanting, will also likely promote herbivory tolerance in rice fields. Ensuring that rice plants have a high tolerance allows natural enemies to build up numbers, as planthopper densities increase during early crop stages [24,80,86], but without causing excessive damage to the plants.

## 5. Conclusions

Rice plants demonstrated susceptibility, resistance and tolerance to the brown planthopper in this study. Under conditions of high planthopper densities (i.e., high nitrogen and excessive insecticide use), plants were both resistant and tolerant to the planthopper, but could not be both susceptible and tolerant, indicating that at least moderate resistance was required for tolerance to the brown planthopper. We found that previously recognized tolerant varieties were also relatively resistant to the planthopper, at low or high soil nitrogen levels. Tolerance was associated with plant size, suggesting that dwarf varieties may be generally non-tolerant to planthopper damage. Moreover, increasing soil nitrogen levels reduced the resistance of rice to the brown planthopper; however, plants with major resistance genes remained relatively more resistant to the planthopper, compared with susceptible varieties. Increasing soil nitrogen levels enhanced tolerance in mainly fast-growing varieties with resistance genes, including varieties with the (uninhibited) *Bph1*-resistance gene. Further research could be directed toward herbivory tolerance to support sustainable and climate-resilient rice production systems.

## Figures and Tables

**Table 1:** Planthopper responses to rice lines under three nitrogen treatments (1 = zero added nitrogen, 2 = 80 Kg ha^−1^, 3 = 150 Kg ha^−1^). Numbers are means ± SEM. Further details are presented in Table S1.

Variety	Nitrogen ^1^	Xylem as Proportion of Total Honeydew ^2^	Development to Adult Stage (Proportion) ^2^	Adult Females (Proportion) ^2^	Nymph Biomass (mg Dry Weight) ^2^	Eggs Per Plant ^2^
IR62	1	**0.89 ± 0.11 ^ef^**	**0.25 ± 0.14 ^a^**	0.75 ± 0.14	**0.48 ± 0.25 ^ab^**	**141.00 ± 31.01 ^a–c^**
	2	**0.24 ± 0.18**	**0.42 ± 0.22**	0.15 ± 0.10	**1.78 ± 0.20**	**111.67 ± 7.22**
	3	**0.73 ± 0.16**	**0.35 ± 0.05**	0.67 ± 0.33	**2.45 ± 0.38**	**144.00 ± 40.25**
ADR52	1	**0.81 ± 0.09 ^c–f^**	**0.69 ± 0.06 ^ab^**	0.53 ± 0.24	**2.06 ± 0.27 ^a–c^**	**93.67 ± 29.63 ^a–c^**
	2	**0.67 ± 0.33**	**0.81 ± 0.10**	0.28 ± 0.15	**1.29 ± 0.11**	**166.33 ± 22.70**
	3	**0.33 ± 0.12**	**0.47 ± 0.14**	0.44 ± 0.29	**1.51 ± 0.25**	**206.33 ± 84.94**
IR60	1	**0.48 ± 0.25 ^c–f^**	0.33 ± 0.33 ^a–c^	1.00 ± 0.00	**1.02 ± 0.34 ^a^**	**110.33 ± 29.69 ^ab^**
	2	**0.79 ± 0.11**	0.62 ± 0.22	0.61 ± 0.20	**1.47 ± 0.45**	**89.00 ± 25.36**
	3	**0.46 ± 0.07**	0.88 ± 0.06	0.47 ± 0.03	**1.78 ± 0.35**	**158.33 ± 38.67**
IR65482-4	1	**0.77 ± 0.13 ^f^**	0.42 ± 0.30 ^a–d^	0.42 ± 0.22	**1.01 ± 0.48 ^ab^**	**47.67 ± 15.77 ^a–d^**
	2	**0.97 ± 0.03**	0.77 ± 0.15	0.28 ± 0.15	**1.39 ± 0.23**	**102.67 ± 16.71**
	3	**0.67 ± 0.16**	0.92 ± 0.08	0.57 ± 0.05	**1.92 ± 0.13**	**180.33 ± 43.84**
Pokkali	1	**0.22 ± 0.10 ^a–e^**	1.00 ± 0.00 ^d^	0.33 ± 0.19	**2.18 ± 0.80 ^a–c^**	**111.67 ± 31.64 ^ab^**
	2	**0.47 ± 0.21**	1.00 ± 0.00	0.17 ± 0.08	**1.16 ± 0.23**	**69.33 ± 25.56**
	3	**0.38 ± 0.14**	1.00 ± 0.00	0.62 ± 0.22	**1.70 ± 0.61**	**157.67 ± 30.07**
Swarnalata	1	**0.94 ± 0.06 ^d–f^**	0.81 ± 0.10 ^b–d^	0.92 ± 0.08	2.61 ± 0.21 ^cd^	**131.00 ± 37.31 ^a–d^**
	2	**0.40 ± 0.31**	0.83 ± 0.17	0.80 ± 0.12	3.15 ± 0.33	**222.33 ± 32.84**
	3	**0.62 ± 0.10**	0.94 ± 0.06	0.45 ± 0.03	3.39 ± 0.21	**173.00 ± 10.79**
Triveni	1	**0.28 ± 0.21 ^a–d^**	0.48 ± 0.08 ^a–d^	1.00 ± 0.00	2.31 ± 0.36 ^a–d^	**58.33 ± 13.53 ^a^**
	2	**0.23 ± 0.03**	0.72 ± 0.15	0.56 ± 0.06	1.99 ± 0.22	**96.00 ± 24.11**
	3	**0.45 ± 0.29**	0.94 ± 0.06	0.34 ± 0.09	2.32 ± 1.46	**100.67 ± 5.36**
IR82396 H	1	**0.32 ± 0.10 ^b–f^**	0.83 ± 0.08 ^b–d^	0.29 ± 0.04	1.68 ± 0.13 ^a–d^	**93.00 ± 29.14 ^a–c^**
	2	**0.44 ± 0.12**	1.00 ± 0.00	0.56 ± 0.12	2.17 ± 0.45	**173.33 ± 13.92**
	3	**0.64 ± 0.10**	1.00 ± 0.00	0.28 ± 0.15	2.05 ± 0.54	**125.33 ± 41.90**
IR82391 H	1	**0.41 ± 0.09 ^a–d^**	0.94 ± 0.06 ^d^	0.87 ± 0.13	4.61 ± 0.06 ^d^	**66.33 ± 14.62 ^a–d^**
	2	**0.30 ± 0.07**	1.00 ± 0.00	0.67 ± 0.09	3.00 ± 0.67	**203.00 ± 64.14**
	3	**0.27 ± 0.06**	1.00 ± 0.00	0.45 ± 0.03	3.18 ± 0.20	**224.67 ± 17.98**
IR46	1	0.28 ± 0.10 ^a–c^	0.78 ± 0.22 ^b–d^	0.42 ± 0.30	2.35 ± 0.46 ^a–d^	**121.67 ± 48.70 ^a–d^**
	2	0.21 ± 0.10	1.00 ± 0.00	0.43 ± 0.03	2.52 ± 0.33	**215.33 ± 37.23**
	3	0.21 ± 0.13	0.85 ± 0.08	0.19 ± 0.10	1.49 ± 0.19	**149.67 ± 35.80**
TKM6	1	0.16 ± 0.07 ^a^	0.87 ± 0.13 ^b–d^	0.33 ± 0.17	1.64 ± 0.28 ^a–d^	**199.67 ± 22.26 ^a–d^**
	2	0.07 ± 0.03	1.00 ± 0.00	0.50 ± 0.14	2.23 ± 0.77	**158.67 ± 58.29**
	3	0.09 ± 0.02	0.92 ± 0.08	0.56 ± 0.29	2.03 ± 0.47	**145.33 ± 8.25**
Utri Rajapan	1	0.35 ± 0.19 ^ab^	1.00 ± 0.00 ^b–d^	0.65 ± 0.05	3.66 ± 0.39 ^a–d^	**216.33 ± 37.43 ^a–d^**
	2	0.12 ± 0.09	1.00 ± 0.00	0.40 ± 0.31	1.77 ± 0.75	**159.33 ± 33.53**
	3	0.13 ± 0.10	0.87 ± 0.13	0.47 ± 0.24	1.95 ± 0.58	**143.00 ± 15.62**
IR40	1	0.39 ± 0.18 ^a–d^	1.00 ± 0.00 ^d^	0.62 ± 0.22	2.89 ± 0.58 ^a–d^	**215.00 ± 29.67 ^a–d^**
	2	0.25 ± 0.04	1.00 ± 0.00	0.67 ± 0.17	3.40 ± 0.51	**207.67 ± 57.78**
	3	0.16 ± 0.07	1.00 ± 0.00	0.63 ± 0.19	2.75 ± 0.47	**110.00 ± 37.04**
ASD7	1	0.39 ± 0.16 ^ab^	0.93 ± 0.07 ^b–d^	0.67 ± 0.33	3.92 ± 1.48 ^cd^	168.00 ± 30.05 ^b–d^
	2	0.15 ± 0.12	0.81 ± 0.19	0.47 ± 0.12	2.48 ± 0.19	189.00 ± 27.00
	3	0.03 ± 0.02	0.79 ± 0.21	0.73 ± 0.18	3.46 ± 0.43	270.00 ± 41.58
IR22	1	0.12 ± 0.03 ^a^	1.00 ± 0.00 ^b–d^	0.39 ± 0.14	1.69 ± 0.17 ^a–d^	263.33 ± 15.43 ^cd^
	2	0.11 ± 0.04	1.00 ± 0.00	0.56 ± 0.08	3.05 ± 0.75	235.33 ± 41.30
	3	0.07 ± 0.02	0.76 ± 0.12	0.89 ± 0.11	3.25 ± 0.41	184.67 ± 3.18
TN1	1	0.09 ± 0.03 ^a^	0.93 ± 0.07 ^b–d^	0.55 ± 0.10	3.15 ± 0.84 ^cd^	234.33 ± 8.09 ^d^
	2	0.01 ± 0.00	0.72 ± 0.19	0.78 ± 0.15	6.28 ± 1.74	271.00 ± 44.88
	3	0.02 ± 0.00	1.00 ± 0.00	0.13 ± 0.13	3.60 ± 1.75	272.00 ± 32.88
F-variety ^3^		10.045 ***	6.636 ***	1.519ns	4.559 ***	5.397 ***
F-nitrogen ^3^		2.547ns	2.448ns	3.551 *	0.024ns	3.496 *
F-interaction ^3^		1.075ns	1.090ns	1.718ns	1.650 *	1.830 *

^1^: 1 = 0 added nitrogen, 2 = 80 Kg N ha^−1^, 3 = 150 Kg N ha^−1^; ^2^: Lowercase letters indicate homogenous variety groups based on Tukey’s LSD test (*p* > 0.05), bold font indicates a variety significantly more resistant than TN1 based on Duncan’s 1-sided test (*p* ≤ 0.05). Varieties are presented in order of most to least resistant based on combined fitness tests; ^3^: ns = no significant effect (*p* > 0.05), * = *p* ≤ 0.05, *** = *p* ≤ 0.001; degrees of freedom (df) = 15,94 for variety, 2,94 for nitrogen, and 30,94 for interaction. Block effects are not reported (df = 1,94).

**Table 2:** Plant growth under varying soil nitrogen levels (1 = zero added nitrogen, 2 = 80 Kg ha^−1^, 3 = 150 Kg ha^−1^) with plant weight loss due to herbivory, and weight lost per unit weight of nymph, as estimated from the Survival bioassays. Numbers are means ± SEM. For further details see Table S2.

Variety	Nitrogen ^1^	Shoot Biomass (g Dry Weight) ^2^	Root Biomass (g Dry Weight) ^2^	Growth Rate (g Dry Weight Day^−1^) ^2^	Weight Loss (g) ^2^	Weight Loss (g mg − Insect^−1^) ^2^
IR82391 H	1	**0.70 ± 0.07 ^a^**	**0.44 ± 0.09 ^a^**	**0.07 ± 0.00 ^a^**	**−0.47 ± 0.32 ^a^**	−0.10 ± 0.07 ^b^
	2	**1.31 ± 0.21**	**0.77 ± 0.10**	**0.12 ± 0.02**	**−0.58 ± 0.26**	−0.25 ± 0.16
	3	**2.34 ± 0.06**	**1.08 ± 0.03**	**0.20 ± 0.00**	**−1.26 ± 0.09**	−0.40 ± 0.02
IR40	1	**0.59 ± 0.04 ^a^**	**0.41 ± 0.04 ^ab^**	**0.04 ± 0.01 ^ab^**	**−0.35 ± 0.06 ^a^**	−0.13 ± 0.04 ^b^
	2	**1.90 ± 0.43**	**1.23 ± 0.94**	**0.19 ± 0.09**	**−1.48 ± 1.18**	−0.37 ± 0.26
	3	**1.96 ± 0.11**	**0.87 ± 0.16**	**0.16 ± 0.01**	**−0.81 ± 0.28**	−0.34 ± 0.14
ASD7	1	1.42 ± 0.46 ^b–f^	0.90 ± 0.14 ^a–e^	0.12 ± 0.04 ^a–c^	−0.94 ± 0.53 ^a–c^	−0.20 ± 0.07 ^b^
	2	2.74 ± 0.82	1.45 ± 0.24	0.19 ± 0.05	−1.48 ± 1.53	−0.52 ± 0.61
	3	3.03 ± 0.52	1.64 ± 0.41	0.24 ± 0.05	−1.58 ± 0.47	−0.44 ± 0.08
IR22	1	**0.90 ± 0.49 ^ab^**	**0.62 ± 0.22 ^ab^**	0.09 ± 0.05 ^a–c^	−0.81 ± 0.95 ^a–c^	−0.55 ± 0.59 ^b^
	2	**1.54 ± 0.31**	**0.88 ± 0.20**	0.15 ± 0.03	−0.16 ± 0.45	−0.17 ± 0.25
	3	**2.93 ± 0.01**	**1.73 ± 0.31**	0.29 ± 0.03	−2.53 ± 0.16	−0.82 ± 0.17
TKM6	1	**1.04 ± 0.13 ^a–d^**	**0.72 ± 0.06 ^ab^**	**0.10 ± 0.01 ^a–c^**	−0.60 ± 0.68 ^ab^	−0.39 ± 0.44 ^b^
	2	**2.03 ± 0.07**	**1.28 ± 0.20**	**0.19 ± 0.02**	−1.76 ± 0.15	−0.99 ± 0.30
	3	**2.36 ± 0.30**	**0.95 ± 0.22**	**0.19 ± 0.03**	−0.75 ± 0.91	−0.51 ± 0.64
Swarnalata	1	1.79 ± 0.24 ^c–f^	1.26 ± 0.17 ^b–e^	0.16 ± 0.02 ^b–d^	−1.55 ± 0.62 ^a–c^	−0.64 ± 0.30 ^ab^
	2	2.91 ± 0.50	1.71 ± 0.29	0.27 ± 0.05	−2.06 ± 0.98	−0.72 ± 0.39
	3	3.86 ± 0.27	1.75 ± 0.21	0.32 ± 0.04	−2.73 ± 0.33	−0.82 ± 0.14
IR65482−4	1	0.71 ± 0.01 ^a–e^	**0.49 ± 0.05 ^a–c^**	0.06 ± 0.00 ^a–c^	−0.27 ± 0.12 ^a–c^	−0.51 ± 0.26 ^ab^
	2	2.13 ± 0.13	**1.14 ± 0.16**	0.18 ± 0.01	−1.06 ± 0.38	−0.72 ± 0.15
	3	3.53 ± 0.43	**1.81 ± 0.17**	0.30 ± 0.00	−2.14 ± 0.39	−1.10 ± 0.14
TN1	1	2.27 ± 0.23 ^d–f^	1.60 ± 0.33 ^c–e^	0.22 ± 0.05 ^cd^	−2.26 ± 0.83 ^a–c^	−1.08 ± 0.70 ^ab^
	2	2.56 ± 0.12	1.50 ± 0.00	0.22 ± 0.01	−1.95 ± 0.41	−0.40 ± 0.19
	3	3.49 ± 0.81	2.58 ± 0.21	0.34 ± 0.07	−3.19 ± 1.49	−0.95 ± 0.50
IR46	1	1.45 ± 0.37 ^b–f^	0.87 ± 0.07 ^a–d^	0.13 ± 0.03 ^a–c^	−1.44 ± 0.35 ^a–c^	−0.64 ± 0.16 ^ab^
	2	3.71 ± 0.34	1.63 ± 0.16	0.31 ± 0.03	−3.02 ± 0.68	−1.22 ± 0.28
	3	2.70 ± 0.43	1.45 ± 0.15	0.22 ± 0.04	−0.88 ± 0.64	−0.58 ± 0.38
ADR52	1	1.79 ± 0.24 ^fg^	1.15 ± 0.27 ^a–e^	0.16 ± 0.04 ^cd^	−1.07 ± 0.11 ^a–c^	−0.54 ± 0.08 ^ab^
	2	3.17 ± 0.23	1.28 ± 0.10	0.23 ± 0.01	−0.64 ± 0.33	−0.52 ± 0.30
	3	5.42 ± 0.14	1.98 ± 0.16	0.40 ± 0.01	−3.13 ± 1.22	−1.93 ± 0.42
IR60	1	0.79 ± 0.31 ^a–e^	0.47 ± 0.17 ^a–d^	0.05 ± 0.02 ^a–c^	−0.41 ± 0.51 ^a–c^	−0.40 ± 0.70 ^ab^
	2	2.40 ± 0.23	1.28 ± 0.28	0.20 ± 0.04	−1.43 ± 0.22	−1.28 ± 0.52
	3	3.35 ± 0.71	2.03 ± 0.55	0.29 ± 0.08	−2.19 ± 1.04	−1.61 ± 1.05
Pokkali	1	1.41 ± 0.12 ^e–g^	0.86 ± 0.01 ^a–d^	0.12 ± 0.02 ^abc^	−1.24 ± 0.22 ^a–c^	−0.69 ± 0.17 ^ab^
	2	3.12 ± 0.95	1.65 ± 0.58	0.22 ± 0.08	−2.00 ± 0.76	−1.70 ± 0.42
	3	4.36 ± 0.16	1.17 ± 0.07	0.32 ± 0.02	−2.13 ± 0.91	−1.10 ± 0.22
Utri Rajapan	1	2.76 ± 0.22 ^g^	1.52 ± 0.18 ^e^	0.24 ± 0.01 ^d^	−2.87 ± 0.06 ^c^	−0.80 ± 0.08 ^ab^
	2	4.45 ± 0.67	2.01 ± 0.04	0.37 ± 0.04	−2.82 ± 0.63	−0.96 ± 0.17
	3	4.84 ± 0.32	3.17 ± 0.49	0.44 ± 0.03	−3.70 ± 0.34	−2.15 ± 0.46
IR62	1	**1.24 ± 0.22 ^a–c^**	0.92 ± 0.27 ^a–d^	0.14 ± 0.03 ^a–c^	−1.67 ± 0.31 ^a–c^	−1.26 ± 0.15 ^ab^
	2	**1.47 ± 0.27**	1.65 ± 0.36	0.20 ± 0.04	−1.96 ± 0.51	−1.18 ± 0.42
	3	**2.72 ± 1.09**	1.85 ± 0.59	0.28 ± 0.10	−4.08 ± 1.45	−1.76 ± 0.68
IR82396 H	1	1.28 ± 0.30 ^b–f^	1.03 ± 0.18 ^a–e^	0.13 ± 0.03 ^cd^	−1.68 ± 0.49^bc^	−1.04 ± 0.36 ^ab^
	2	3.56 ± 0.57	2.21 ± 0.77	0.37 ± 0.07	−4.90 ± 1.45	−2.45 ± 0.98
	3	3.46 ± 0.64	1.79 ± 0.32	0.31 ± 0.06	−2.67 ± 0.71	−1.35 ± 0.37
Triveni	1	1.81 ± 0.28 ^e–g^	1.47 ± 0.27 ^de^	0.16 ± 0.02 ^cd^	−1.98 ± 0.09 ^a–c^	**−0.89 ± 0.11 ^a^**
	2	2.76 ± 0.14	2.28 ± 0.03	0.26 ± 0.02	−2.34 ± 0.15	**−1.22 ± 0.22**
	3	4.23 ± 0.50	2.37 ± 0.39	0.40 ± 0.06	−4.07 ± 0.98	**−4.55 ± 2.44**
F−variety ^3^		11.225 ***	6.608 ***	6.174 ***	3.655 ***	2.974 ***
F−nitrogen ^3^		99.596 ***	37.459 ***	66.639 ***	10.269 ***	6.327 ***
F−interaction ^3^		1.365ns	1.029ns	1.136ns	1.171ns	1.323ns

^1^: 1 = 0 added nitrogen, 2 = 60 Kg N ha^−1^, 3 = 150 Kg N ha^−1^; ^2^: Lowercase letters indicate homogenous variety groups based on Tukey’s LSD test (*p* > 0.05), bold font indicates a variety significantly more resistant than TN1 based on Duncan’s 1-sided test (*p* ≤ 0.05). Varieties are presented in order of lowest to highest losses of plant biomass per unit insect weight; ^3^: ns = no significant effect (*p* > 0.05), *** = *p* ≤ 0.001; degrees of freedom (df) = 15,94 for variety, 2,94 for nitrogen, and 30,94 for interaction. Block effects are not reported (df = 1,94).

**Table 3:** Best-fit linear models for weight loss to plants under three different nitrogen regimes and plant weight loss per mg of planthopper (Figure 2).

Model	Slope	Intercept	R^2^	F_1,15_^1^
Weight loss				
0 added nitrogen	−13.007	0.385	0.90	127.269 ***
80 Kg N ha^−1^	−13.463	1.242	0.74	38.847 ***
150 Kg N ha^−1^	−11.634	1.053	0.71	33.592 ***
Weight loss per mg insect				
0 added nitrogen	−4.038	−0.115	0.43	10.559 **
80 Kg N ha^−1^	−5.244	0.288	0.41	9.219 **
150 Kg N ha^−1^	−7.417	1.014	0.69	30.578 ***

^1^: *** = *p* ≤ 0.001, ** = *p* ≤ 0.01.

## Data Availability

The data presented in this study are available on request from the corresponding author.

## Supplementary Materials

The following are available online at https://www.mdpi.com/article/10.3390/insects12110989/s1, Table S1: Planthopper condition after 15 days of exposure to rice varieties in the Survival bioassay under three levels of soil nitrogen, Table S2: Growth parameters for planthopper infested and control, non-infested plants during the Survival bioassay under three levels of soil nitrogen.
