# Supplementary material for: Nitrogenous Fertilizer Reduces Resistance but Enhances Tolerance to the Brown Planthopper in Fast-Growing, Moderately Resistant Rice
Source: Insects. 2021 Nov 3;12(11):989. doi: 10.3390/insects12110989 (PMC8621593; doi:10.3390/insects12110989)
Supplement: Supplementary file 1 [file insects-12-00989-s001.zip › insects-1439511-supplementary.pdf]

Supplementary Information

Table S1: Planthopper condition after 15 days of exposure to rice varieties in the Survival bioassay under three levels of soil nitrogen. Means ± SEM are presented. Further details are presented in Table 1.

Variety[1]	Resistance sources[2]	Reaction[3]	Nitrogen[4]	Development (Proportion at each stage)[5]				Brachypterous[5]		Nymph survival (proportion)[5]
				3rd instar	4th instar	5th instar	Adult	Female	Male	
ADR52	*BPH25, BPH26* + unknown	R + T	1.00	0.00±0.00	0.24±0.04	0.07±0.07	0.69±0.06 ^{ab}	1.00±0.00	0.39±0.20 ^{a}	0.54±0.08
			2.00	0.00±0.00	0.08±0.08	0.11±0.11	0.81±0.10	1.00±0.00	0.33±0.33	0.42±0.04
			3.00	0.00±0.00	0.11±0.11	0.42±0.13	0.47±0.14	1.00±0.00	0.25±0.14	0.42±0.04
ASD7	*bph2*	S	1.00	0.00±0.00	0.00±0.00	0.07±0.07	0.93±0.07 ^{bcd}	1.00±0.00	0.33±0.00 ^{a}	0.50±0.07
			2.00	0.07±0.07	0.07±0.07	0.04±0.04	0.81±0.19	1.00±0.00	0.00±0.00	0.59±0.16
			3.00	0.00±0.00	0.00±0.00	0.21±0.21	0.79±0.21	1.00±0.00	0.00±0.00	0.75±0.13
IR22	None	S	1.00	0.00±0.00	0.00±0.00	0.00±0.00	1.00±0.00 ^{bcd}	1.00±0.00	0.22±0.11 ^{a}	0.46±0.04
			2.00	0.00±0.00	0.00±0.00	0.00±0.00	1.00±0.00	1.00±0.00	0.50±0.29	0.54±0.08
			3.00	0.00±0.00	0.00±0.00	0.24±0.24	0.76±0.12	0.92±0.08	0.00±0.00	0.50±0.07
IR40	*Bph1*	S	1.00	0.00±0.00	0.00±0.00	0.00±0.00	1.00±0.00 ^{d}	1.00±0.00	0.00±0.00 ^{a}	0.50±0.07
			2.00	0.00±0.00	0.00±0.00	0.00±0.00	1.00±0.00	1.00±0.00	0.25±0.15	0.50±0.00
			3.00	0.00±0.00	0.00±0.00	0.00±0.00	1.00±0.00	1.00±0.00	0.00±0.00	0.46±0.11
IR46	*Bph1*	MR + T	1.00	0.00±0.00	0.00±0.00	0.22±0.22	0.78±0.22 ^{bcd}	1.00±0.00	0.50±0.29 ^{a}	0.46±0.04
			2.00	0.00±0.00	0.00±0.00	0.00±0.00	1.00±0.00	0.83±0.17	0.28±0.15	0.58±0.04
			3.00	0.00±0.00	0.00±0.00	0.15±0.08	0.85±0.08	1.00±0.00	0.33±0.33	0.50±0.07
IR60	*Bph3/BPH32*	MR	1.00	0.23±0.15	0.43±0.23	0.00±0.00	0.33±0.33^{abc}	1.00±0.00	0.50±0.29 ^{a}	0.42±0.15
			2.00	0.17±0.17	0.00±0.00	0.22±0.12	0.62±0.22	0.92±0.08	0.50±0.29	0.71±0.15
			3.00	0.00±0.00	0.00±0.00	0.12±0.06	0.88±0.06	1.00±0.00	0.00±0.00	0.54±0.15
IR62	*Bph3/BPH32*	R + T	1.00	0.00±0.00	0.42±0.30	0.33±0.33	0.25±0.14 ^{a}	1.00±0.00	1.00±0.00 ^{b}	0.38±0.13
			2.00	0.00±0.00	0.39±0.31	0.19±0.19	0.42±0.22	1.00±0.00	0.44±0.29	0.46±0.18
			3.00	0.00±0.00	0.43±0.12	0.22±0.22	0.35±0.05	1.00±0.00	1.00±0.00	0.58±0.04
IR65482-4	*Bph10*	MR + T	1.00	0.00±0.00	0.50±0.29	0.08±0.08	0.42±0.30 ^{abcd}	1.00±0.00	0.00±0.00 ^{a}	0.50±0.00
			2.00	0.00±0.00	0.00±0.00	0.23±0.15	0.77±0.15	1.00±0.00	0.33±0.33	0.42±0.11
			3.00	0.00±0.00	0.00±0.00	0.08±0.08	0.92±0.08	1.00±0.00	0.50±0.29	0.50±0.00
IR82391 H	*Bph1*	S	1.00	0.00±0.00	0.08±0.08	0.08±0.08	0.83±0.08 ^{bcd}	1.00±0.00	0.17±0.17 ^{a}	0.54±0.04
			2.00	0.00±0.00	0.00±0.00	0.00±0.00	1.00±0.00	0.89±0.11	0.00±0.00	0.44±0.04
			3.00	0.00±0.00	0.00±0.00	0.00±0.00	1.00±0.00	1.00±0.00	0.42±0.30	0.46±0.04

IR82396 H	None	S-MR + T	1.00	0.00±0.00	0.00±0.00	0.06±0.06	0.94±0.06 ^{d}	1.00±0.00	0.50±0.00 ^{a}	0.62±0.07
			2.00	0.00±0.00	0.00±0.00	0.00±0.00	1.00±0.00	1.00±0.00	0.25±0.14	0.58±0.04
			3.00	0.00±0.00	0.00±0.00	0.00±0.00	1.00±0.00	1.00±0.00	0.11±0.11	0.58±0.04
Pokkali	*Bph9*	R	1.00	0.00±0.00	0.00±0.00	0.00±0.00	1.00±0.00 ^{d}	1.00±0.00	0.33±0.33 ^{a}	0.38±0.00
			2.00	0.00±0.00	0.00±0.00	0.00±0.00	1.00±0.00	1.00±0.00	0.11±0.11	0.42±0.08
			3.00	0.00±0.00	0.00±0.00	0.00±0.00	1.00±0.00	1.00±0.00	0.28±0.15	0.42±0.15
Swarnalata	*Bph6*	MR	1.00	0.00±0.00	0.00±0.00	0.19±0.19	0.81±0.10 ^{bcd}	1.00±0.00	0.00±0.00 ^{a}	0.46±0.04
			2.00	0.00±0.00	0.00±0.00	0.17±0.17	0.83±0.17	1.00±0.00	0.00±0.00	0.58±0.04
			3.00	0.00±0.06	0.00±0.00	0.00±0.00	0.94±0.06	1.00±0.00	0.41±0.05	0.62±0.07
TKM6	*Bph1*	S	1.00	0.00±0.00	0.00±0.00	0.13±0.13	0.87±0.13 ^{bcd}	1.00±0.00	0.32±0.19 ^{a}	0.46±0.11
			2.00	0.00±0.00	0.00±0.00	0.00±0.00	1.00±0.00	1.00±0.00	0.11±0.11	0.42±0.08
			3.00	0.00±0.00	0.00±0.00	0.08±0.08	0.92±0.08	1.00±0.00	0.00±0.00	0.42±0.08
TN1	None	S	1.00	0.00±0.00	0.00±0.00	0.07±0.07	0.93±0.07 ^{bcd}	1.00±0.00	0.28±0.15 ^{ab}	0.58±0.04
			2.00	0.00±0.00	0.00±0.00	0.28±0.28	0.72±0.19	1.00±0.00	0.75±0.14	0.76±0.08
			3.00	0.00±0.00	0.00±0.00	0.00±0.00	1.00±0.00	1.00±0.00	0.36±0.22	0.46±0.11
Triveni	unknown	MR + T	1.00	0.00±0.00	0.11±0.11	0.41±0.05	0.48±0.08 ^{abcd}	0.61±0.20	0.00±0.00 ^{a}	0.50±0.07
			2.00	0.06±0.06	0.08±0.08	0.14±0.14	0.72±0.15	1.00±0.00	0.17±0.17	0.54±0.11
			3.00	0.00±0.00	0.00±0.00	0.06±0.06	0.94±0.06	1.00±0.00	0.58±0.30	0.46±0.15
Utri Rajapan	unknown	MR + T	1.00	0.00±0.00	0.00±0.00	0.00±0.00	1.00±0.00 ^{bcd}	1.00±0.00	0.00±0.00 ^{a}	0.58±0.04
			2.00	0.00±0.00	0.00±0.00	0.00±0.00	1.00±0.00	1.00±0.00	0.00±0.00	0.42±0.15
			3.00	0.00±0.00	0.13±0.13	0.00±0.00	0.87±0.13	1.00±0.00	0.33±0.33	0.54±0.04
F-variety							6.636***	1.541ns	3.496***	1.498ns
F-nitrogen							2.448ns	0.723ns	0.218ns	0.843ns
F-interaction							1.090ns	1.991**	1.501ns	0.970ns

1: Varieties are presented in alphabetical order

2: Genes are based on published records as indicated in the main text

3: Reactions are based on insect responses to plants from this study and corresponding damage to plant, also based on this study

4: 1 = 0 added nitrogen, 2 = 60 Kg N ha^{-1}, 3 = 150 Kg N ha^{-1}

5: Lowercase letters indicate homogenous variety groups based on Tukey's LSD test ($P > 0.05$); ns = no significant effect ($P > 0.05$), ** = $P \leq 0.01$; *** = $P \leq 0.001$; degrees of freedom (df) = 15,94 for variety, 2,94 for nitrogen, and 30,94 for interaction.

Table S2: Growth parameters for planthopper infested and control, non-infested plants during the Survival bioassay under three levels of soil nitrogen. Means ± SEM are presented. Further details are presented in Table 2. Data analyses are presented in the main text.

Variety[1]	Nitrogen[2]	Damage rating (SES score)	Reaction[3]	30 DAS shoot weight (g dry weight)	30 DAS root weight (g dry weight)	45 DAS shoot weight (g dry weight)	45 DAS root weight (g dry weight)	30 DAS non-infested plant weight (g dry weight)	45 DAS non-infested plant weight (g dry weight)	Growth rate of non-infested plants (g day-1)	45 DAS infested plant weight (g dry weight)	Growth rate of infested plants (g day-1)
ADR52	1	1.33±0.88	R	0.43±0.14	0.18±0.06	1.79±0.24	1.15±0.27	0.61±0.20	3.37±0.47	0.16±0.04	1.87±0.58	0.08±0.05
	2	1.00±0.00	R	0.64±0.17	0.31±0.06	3.17±0.23	1.28±0.10	0.95±0.22	5.12±0.34	0.23±0.01	3.81±0.06	0.19±0.02
	3	2.33±1.33	R	0.88±0.14	0.45±0.08	5.42±0.14	1.98±0.16	1.33±0.22	7.92±0.30	0.40±0.01	4.27±1.49	0.20±0.08
ASD7	1	5.67±0.67	S	0.35±0.14	0.23±0.09	1.42±0.46	0.90±0.14	0.59±0.22	3.38±0.61	0.12±0.04	1.38±0.50	0.05±0.02
	2	6.33±0.33	S	0.80±0.28	0.54±0.26	2.74±0.82	1.45±0.24	1.34±0.51	5.44±1.06	0.19±0.05	2.71±0.49	0.09±0.06
	3	5.67±0.67	S	0.71±0.18	0.42±0.05	3.03±0.52	1.64±0.41	1.14±0.23	5.31±0.92	0.24±0.05	3.09±0.54	0.13±0.02
IR22	1	6.33±0.33	S	0.08±0.04	0.05±0.02	0.90±0.49	0.62±0.22	0.13±0.06	0.78±0.71	0.09±0.05	0.70±0.32	0.04±0.02
	2	6.33±0.33	S	0.13±0.07	0.07±0.02	1.54±0.31	0.88±0.20	0.20±0.09	1.65±0.51	0.15±0.03	2.26±0.11	0.14±0.00
	3	6.67±0.33	S	0.22±0.06	0.15±0.04	2.93±0.01	1.73±0.31	0.37±0.10	4.70±0.29	0.29±0.03	2.13±0.36	0.12±0.03
IR40	1	6.00±1.00	S	0.21±0.07	0.12±0.02	0.59±0.04	0.41±0.04	0.34±0.08	1.00±0.08	0.04±0.01	0.65±0.04	0.02±0.01
	2	6.33±0.33	S	0.15±0.03	0.05±0.01	1.90±0.43	1.23±0.94	0.20±0.03	2.24±1.36	0.19±0.09	1.65±0.18	0.10±0.01
	3	7.00±0.00	S	0.23±0.09	0.18±0.04	1.96±0.11	0.87±0.16	0.41±0.12	2.29±0.27	0.16±0.01	2.02±0.01	0.11±0.01
IR46	1	5.67±1.33	S	0.15±0.05	0.17±0.03	1.45±0.37	0.87±0.07	0.32±0.05	1.61±0.40	0.13±0.03	0.88±0.42	0.04±0.03
	2	4.33±0.67	S	0.49±0.17	0.14±0.02	3.71±0.34	1.63±0.16	0.63±0.19	5.09±0.33	0.31±0.03	2.32±0.46	0.11±0.04
	3	5.00±1.15	S	0.53±0.20	0.27±0.15	2.70±0.43	1.45±0.15	0.80±0.35	3.80±0.28	0.22±0.04	3.27±0.86	0.16±0.06
IR60	1	3.67±1.33	MR	0.27±0.12	0.21±0.09	0.79±0.31	0.47±0.17	0.48±0.21	0.63±0.47	0.05±0.02	0.85±0.11	0.02±0.02
	2	3.67±0.67	MR	0.34±0.06	0.33±0.09	2.40±0.23	1.28±0.28	0.67±0.13	3.70±0.47	0.20±0.04	2.25±0.37	0.11±0.03
	3	3.00±0.00	MR	0.62±0.03	0.36±0.05	3.35±0.71	2.03±0.55	0.98±0.06	7.82±1.26	0.29±0.08	3.18±0.37	0.15±0.02
IR62	1	1.33±0.88	R	0.06±0.01	0.06±0.00	1.24±0.22	0.92±0.27	0.12±0.01	3.04±0.47	0.14±0.03	0.49±0.17	0.02±0.01
	2	0.67±0.33	R	0.11±0.02	0.05±0.01	1.47±0.27	1.65±0.36	0.16±0.02	3.30±0.58	0.20±0.04	1.16±0.50	0.07±0.03
	3	0.33±0.33	R	0.31±0.00	0.08±0.02	2.72±1.09	1.85±0.59	0.39±0.02	5.55±1.44	0.28±0.10	0.49±0.02	0.01±0.00
IR65482-4	1	4.33±0.67	S	0.17±0.04	0.16±0.03	0.71±0.01	0.49±0.05	0.34±0.02	1.24±0.05	0.06±0.00	0.94±0.08	0.04±0.01
	2	1.67±0.67	R	0.32±0.04	0.23±0.02	2.13±0.13	1.14±0.16	0.55±0.06	3.18±0.21	0.18±0.01	2.21±0.20	0.11±0.01
	3	3.00±0.00	MR	0.58±0.26	0.28±0.09	3.53±0.43	1.81±0.17	0.86±0.34	4.75±0.37	0.30±0.00	3.20±0.51	0.16±0.03
IR82391 H	1	7.00±0.00	S	0.18±0.05	0.12±0.02	1.28±0.30	1.03±0.18	0.30±0.07	1.96±0.47	0.13±0.03	0.63±0.03	0.02±0.01
	2	5.67±0.67	S	0.15±0.03	0.07±0.02	3.56±0.57	2.21±0.77	0.22±0.05	7.55±1.16	0.37±0.07	0.87±0.30	0.04±0.02
	3	6.33±0.33	S	0.35±0.25	0.20±0.06	3.46±0.64	1.79±0.32	0.55±0.30	6.35±0.86	0.31±0.06	2.58±0.15	0.14±0.02

IR82396 H	1	7.00±0.00	S	0.02±0.01	0.02±0.01	0.70±0.07	0.44±0.09	0.04±0.01	1.14±0.03	0.07±0.00	0.67±0.29	0.04±0.02
	2	5.00±1.15	S	0.18±0.02	0.07±0.01	1.31±0.21	0.77±0.10	0.25±0.01	1.61±0.24	0.12±0.02	1.49±0.18	0.08±0.01
	3	3.00±0.00	MR	0.13±0.01	0.36±0.03	2.34±0.06	1.08±0.03	0.49±0.04	3.52±0.09	0.20±0.00	2.16±0.01	0.11±0.00
Pokkali	1	6.00±1.00	S	0.29±0.10	0.18±0.03	1.41±0.12	0.86±0.01	0.47±0.10	2.24±0.13	0.12±0.02	1.02±0.10	0.04±0.00
	2	1.33±0.88	R	0.92±0.30	0.50±0.14	3.12±0.95	1.65±0.58	1.42±0.43	3.54±1.53	0.22±0.08	2.77±0.82	0.09±0.04
	3	1.33±0.88	R	0.50±0.15	0.27±0.06	4.36±0.16	1.17±0.07	0.78±0.20	5.89±0.19	0.32±0.02	3.40±0.89	0.17±0.05
Swarnalata	1	3.00±1.15	MR	0.38±0.10	0.24±0.06	1.79±0.24	1.26±0.17	0.62±0.15	2.31±0.41	0.16±0.02	1.49±0.47	0.06±0.03
	2	3.67±0.67	MR	0.46±0.04	0.18±0.04	2.91±0.50	1.71±0.29	0.63±0.06	3.26±0.79	0.27±0.05	2.55±0.52	0.13±0.03
	3	4.33±0.67	S	0.38±0.10	0.39±0.25	3.86±0.27	1.75±0.21	0.77±0.35	5.77±0.48	0.32±0.04	2.88±0.24	0.14±0.04
TKM6	1	5.67±1.33	S	0.18±0.04	0.16±0.03	1.04±0.13	0.72±0.06	0.34±0.07	1.46±0.18	0.10±0.01	1.17±0.68	0.06±0.04
	2	5.00±0.00	S	0.37±0.15	0.15±0.05	2.03±0.07	1.28±0.20	0.52±0.20	3.61±0.16	0.19±0.02	1.55±0.02	0.07±0.01
	3	5.00±1.15	S	0.30±0.02	0.15±0.03	2.36±0.30	0.95±0.22	0.45±0.04	4.20±0.53	0.19±0.03	2.56±0.42	0.14±0.03
TN1	1	5.00±0.00	S	0.45±0.16	0.19±0.07	2.27±0.23	1.60±0.33	0.64±0.22	3.33±0.78	0.22±0.05	1.61±0.34	0.06±0.01
	2	6.67±0.33	S	0.46±0.09	0.30±0.06	2.56±0.12	1.50±0.00	0.76±0.08	4.10±0.12	0.22±0.01	2.11±0.51	0.09±0.04
	3	6.67±0.33	S	0.63±0.16	0.30±0.09	3.49±0.81	2.58±0.21	0.93±0.24	5.04±1.00	0.34±0.07	2.88±0.62	0.13±0.03
Triveni	1	3.67±1.76	MR	0.51±0.05	0.35±0.01	1.81±0.28	1.47±0.27	0.86±0.04	3.81±0.35	0.16±0.02	1.30±0.29	0.03±0.02
	2	3.67±0.67	MR	0.66±0.15	0.47±0.06	2.76±0.14	2.28±0.03	1.13±0.22	4.72±0.17	0.26±0.02	2.70±0.04	0.10±0.01
	3	3.00±0.00	MR	0.34±0.07	0.32±0.08	4.23±0.50	2.37±0.39	0.66±0.13	4.92±0.88	0.40±0.06	2.53±0.11	0.12±0.01
Utri Rajapan	1	3.00±0.00	MR	0.52±0.08	0.23±0.01	2.76±0.22	1.52±0.18	0.75±0.08	4.36±0.04	0.24±0.01	1.41±0.10	0.04±0.01
	2	3.00±0.00	MR	0.56±0.14	0.32±0.09	4.45±0.67	2.01±0.04	0.89±0.22	7.54±0.63	0.37±0.04	3.65±0.13	0.18±0.02
	3	3.00±0.00	MR	0.92±0.18	0.44±0.11	4.84±0.32	3.17±0.49	1.36±0.27	8.56±0.76	0.44±0.03	4.31±0.42	0.20±0.01

1: Varieties are presented in alphabetical order

2: 1 = 0 added nitrogen, 2 = 60 Kg N ha^{-1}, 3 = 150 Kg N ha^{-1}

3: Reactions are based on standard evaluation system (SES) damage ratings to seedlings in the bioassay. Because plants were harvested before any plants had died, we assigned reactions as follows: < 3 = resistant (R), 3-4 = moderately resistant (MR), and > 4 = susceptible.
